# Efficient fermentation of an improved synthetic grape must by enological and laboratory strains of *Saccharomyces cerevisiae*

**DOI:** 10.1186/s13568-014-0016-0

**Published:** 2014-04-01

**Authors:** Tiago Viana, Maria C Loureiro-Dias, Catarina Prista

**Affiliations:** 1 Centro de Botânica Aplicada à Agricultura, Instituto Superior de Agronomia, Universidade de Lisboa, Lisboa, Portugal

**Keywords:** Synthetic grape must, Natural grape must, Wine fermentation, BY auxotrophic mutant serie

## Abstract

Grape must or freshly pressed grape juice is a complex chemical matrix that impacts the efficiency of yeast fermentation. The composition of natural grape must (NGM) can be variable; thus, to ensure reproducibility, a synthetic grape must (SGM) with defined composition is commonly used. The aim of this work was to create conditions to advance the use of *Saccharomyces cerevisiae* laboratory strains for wine fermentation studies, considering previous results obtained for enological strains fermenting NGM under simulated winery conditions. We designed a new SGM formulation, ISA-SGM, by introducing specific modifications to a commonly used formulation, putting together previous reports. We added glucose and fructose in equal amounts (125 g/l) and 50 parts per million (ppm) sulfur dioxide (SO_2,_ corresponding to standard enological treatment), and we optimized the concentrations of malic acid (3 g/l), citric acid (0.3 g/l), and tartaric acid (3 g/l). Using ISA-SGM, we obtained similar fermentative profiles for the wine strain ISA1000, the prototrophic strain S288C, and its auxotrophic derivative BY4741. In this case, the concentrations of supplements were optimized to 120 mg/l L-uracil, 80 mg/l L-methionine, 400 mg/l L-leucine, and 100 mg/l L-histidine. All these strains tested in ISA-SGM presented a similar fermentative performance as ISA1000 in NGM. ISA-SGM formulation is a promising new tool to allow the use of the auxotrophic BY strains in the detailed assessment of the alcoholic fermentation process under simulated winery conditions, and it provides a foundation to extract relevant physiological conclusions in future research on enological yeast traits.

## Introduction

*Saccharomyces cerevisiae* plays a well-established and fundamental role in the complex process of winemaking. The prevalence of *S. cerevisiae* strains during grape must fermentation has been attributed to various factors, including strong fermentation capacity, high resistance to ethanol, and osmotolerance (Pretorius [2000]). However, the physiology of *S. cerevisiae* also contributes to a chronic problem affecting the wine industry - the occurrence of stuck or sluggish (i.e. incomplete or delayed) fermentations that stop or slow down well before sugar exhaustion (Malherbe et al. [2007]). Stuck fermentations are predominantly caused by the harmful effects of ethanol and other stress factors (Malherbe et al. [2007]; Santos et al. [2008]). Genes involved in tolerance to these factors could be targets for yeast genetic engineering to improve fermentation efficiency and to control the production of wine, at least as far as yeast performance is involved (Fleet [2008]).

To elucidate genetic pathways relevant to winemaking, researchers have screened strains with deletion or overexpression of specific genes (Gómez-Pastor et al. [2010]; López-Malo et al. [2012]; Teixeira et al. [2009]). However, the application of this strategy to wine strains has been impaired by the heterogeneity of industrial yeast genomes, which present frequently polyploidy or aneuploidy. These genomes often display heterozygosity, single nucleotide polymorphisms, strain-specific open reading frames, and localized variations in gene copy number (Borneman et al. [2011a,b]; Bradbury et al. [2006]; Pretorius [2000]). This variability makes it difficult to construct single mutant libraries and to complete whole-genome screening in these strains. One strategy to understand the genetics of industrial wine yeasts is to develop genetically tractable versions of these commercial strains (Borneman et al. [2008]; Engel and Cherry [2013]; Walker et al. [2003,2005]). A single-gene deletion library is currently being developed in a haploid derivative of a wine yeast (Tran et al. [2010]).

*S. cerevisiae* S288C is well-characterized and a commonly used laboratory strain that is the foundation of the single deletion mutant libraries (BY4741, BY4742, and BY4743) available at Euroscarf (Brachmann et al. [1998]; Winzeler et al. [1999]). These single-gene deletion and overexpression strains are a very powerful research tool for elucidating genetic pathways. However, S288C has been reported as a poorly fermenting strain when compared to industrial wine yeast strains (Harsch et al. [2010]; Pizarro et al. [2007]), even with amino acid supplementation (Harsch et al. [2010]). The inferior fermentation ability is even more evident in the S288C-derived BY strains used in the systematic gene deletion project (Hanscho et al. [2012]; Harsch et al. [2010]). While it would be preferable to use wild strains for wine fermentation studies, mutants based on these strains are not yet available. S288C-derived BY strains are auxotrophic for several amino acids and uracil; thus, auxotrophic supplements must be added, appropriate concentrations of which have been determined by considering the composition of the final biomass and the type of energy metabolism (Pronk [2002]). Auxotrophic supplementation is also necessary when using a complex medium, such as Yeast extract Peptone Dextrose (YPD) or grape must (Corbacho et al. [2011]; Hanscho et al. [2012]; Harsch et al. [2010]). The optimal concentration of these supplements is still under debate. In addition to supplements for auxotrophy, other nutrients are required for the optimal growth and metabolic performances of S288C-derived BY strains. These nutrients include inositol, biotin, and mixtures of preferred amino acids (Çakar et al. [1999]; Hanscho et al. [2012]). The amounts and types of these supplements can trigger modifications of energy metabolism, protein expression, final biomass, survival capacity, and stress response (Brauer et al. [2008]; Çakar et al. [1999]; Görgens et al. [2005]).

Grape must is a complex chemical matrix that depends on grape variety, ripeness stage, terroir characteristics, climate, and viticultural factors (Ribereau-Gayon et al. [2006]). While transforming grape must into wine, yeast converts sugars to ethanol and produces various other compounds, which add new degrees of complexity and variability to the original matrix (Lambrechts and Pretorius [2000]). The use of natural grape must (NGM) in the laboratory under simulated enological conditions is the best experimental approach and most precise way to study how wine yeast cope with stress conditions in the winery environment (Rossouw et al. [2012]). However, considering the complexity and variability of NGM and the scientific requirement for reproducible data, researchers tend to use synthetic grape must (SGM) that has a known composition. Using SGM also allows researchers to avoid mutant phenotypes from being masked due to the presence of unknown substrates, especially when testing auxotrophic deletion mutants.

Several base formulations of SGM have been published that vary in the composition and concentration of their components (Bely et al. [1990]; Ciani and Ferraro [1996]; Riou et al. [1997]). The most widely used SGM is MS300, a modified version of the synthetic grape juice first described by Bely et al. ([1990]). Several authors have introduced modifications to the MS300 composition. For example, researchers have replaced glucose with equimolar concentrations of glucose and fructose (100 and 150 g/l) as energy and carbon sources (Marullo et al. [2004]), varied the amount of SO_2_ (Nardi et al. [2010]), removed anaerobic growth factors (Gutiérrez et al. [2012]), varied the concentrations of malic and citric acids (Albertin et al. [2011]; Salmon and Barre [1998]), and added tartaric acid (Albertin et al. [2011]; Marullo et al. [2004]). These modifications were introduced to make the SGM formulation more similar to that of NGM, and to optimize the media for optimal fermentation performance of laboratory strains (Harsch et al. [2010]; Rossouw and Bauer [2009]). Nonetheless, none of these formulations has been completely successful.

In the present work, we tested the ability of two closely related laboratory *S. cerevisiae* strains (S288C and BY4741) to ferment ISA-SGM under conditions similar to those found in wineries. To validate the use of the auxotrophic haploid strain BY4741 (Brachmann et al. [1998]) as a model for wine yeast fermentation studies, the results obtained with this strain were compared with the behavior of an enological yeast strain under the same conditions. We combined suggestions made by some authors improving the formulation of a modified SGM, in which BY4741 can ferment with a comparable performance to that in NGM. This work aims to establish standard conditions for an efficient fermentation using BY4741 single mutation derivative strains to extract relevant physiological data on their fermentative performance in simulated wine fermentation studies.

## Materials and methods

### Yeast strains

The diploid *S. cerevisiae* S288C prototrophic strain and its isogenic auxotrophic haploid strain *S. cerevisiae* BY4741 (MAT**a***his3D1 leu2D0 met15D0; ura3D0* (Brachmann et al. [1998])) were obtained from Euroscarf (http://web.uni-frankfurt.de/fb15/mikro/euroscarf/index.html). In addition, an enological *S. cerevisiae* strain (ISA1000), isolated from a commercial active dry yeast starter (FERMIVIN®), was obtained from the Instituto Superior de Agronomia (ISA) yeast culture collection. Stock cultures were maintained at 4°C on YPD agar (1% yeast extract, 2% peptone, 2% glucose, 2% agar). Yeast strains were stored at −80°C in 15% glycerol and streaked onto YPD plates if necessary.

### Media and fermentation conditions

#### Fermentative media

First fermentations were carried out in synthetic grape must MS300 (Salmon and Barre [1998]), which was modified as described by Rossouw and Bauer ([2009]) and designated as RB-SGM. Briefly, glucose and fructose (125 g/l each) were used as carbon and energy sources. The available nitrogen was 300 mg/l, provided by 460 mg/l NH_4_Cl (corresponding to 120 mg/l of nitrogen) and by a mixture of 19 amino acids (corresponding to 180 mg/l of nitrogen). Ergosterol (15 mg/l) and sodium oleate (5 mg/l) in 1 ml of Tween 80/ethanol (1:1, v/v) were added as anaerobic growth factors, and the pH was adjusted to 3.3 using NaOH. Concentrated solutions of each compound were prepared, filtered through 0.22-μm nitrocellulose membranes (Millipore filter, type GSWP), and added in adequate amounts before inoculation.

When indicated, RB-SGM was modified by varying the concentrations of citric, malic, and tartaric acids, to mimic the normal concentrations of these acids commonly found in must at grape maturity (Ribereau-Gayon et al. [2006]). SO_2_ was adjusted to a final concentration of 50 parts per million (ppm) by adding potassium metabisulfite, as in standard enological treatments. When necessary, auxotrophic supplements were added at concentrations of 60 mg/l L-uracil, 200 mg/l L-leucine, 50 mg/l L-histidine, and 40 mg/l L-methionine (1×) or twice these concentrations (2×). The first values (1×) approximately correspond to those recommended by Pronk (Pronk [2002]), accounting for the biomass content and the expected final biomass obtained during wine fermentation (Viana et al. [2012]). For comparison, fermentation of NGM from white grapes of the Arinto variety collected from the ISA vineyards, supplemented with SO_2_ (50 ppm), was carried out as described previously (Viana et al. [2012]). Whenever necessary, the appropriate auxotrophic supplements indicated above were added to NGM.

#### Fermentative conditions

Yeast inoculation was standardized at 10^6^ cells/ml in 80 ml of liquid medium in 100 ml Erlenmeyer flasks with a cotton cap (Viana et al. [2012]). All fermentations were carried out at 25°C with very low orbital shaking (120 rpm) in a water bath (D-3162 Kottermann type 3047, West Germany). Fermentation progress was monitored by estimating the glucose concentration. After glucose exhaustion, samples were periodically collected for estimating fructose concentration in supernatants after removing the cells by centrifugation (12,000 × *g*, 3 min, 4°C). Fermentation was considered complete when the Portuguese legal maximum limit for residual sugars in wine was reached (≤ 2 g/l).

### Growth monitoring

Yeast growth was monitored by measuring optical densities at 640 nm (OD_640nm_) in an Ultrospec 2100 pro UV-visible (Amersham Biosciences®) spectrophotometer. Growth data were analyzed using the DMFit software available on the Combase website (http://www.combase.cc/index.php/en/). Growth data were fit to the model proposed by Baranyi and Roberts ([1994]) to obtain lag times and specific growth rates of each fermentation assay. Viability was determined throughout fermentation by counting colony forming units (CFU) on YPD solid medium, after 2–3 days of incubation at 28°C.

### Analytical techniques

Dry biomass data were determined by filtering 1 ml of cell suspension through preweighted Whatman membrane filters (pore diameter of 0.2 μm). The filters were rapidly washed with 10 ml of distilled water, dried at 80°C for 24 h, and weighed. Duplicate determinations varied by less than 5%.

The glucose concentration was estimated with a rapid detection assay using a commercially available dipstick (Diabur-Test 5000; Boehringer, Mannheim, Germany) commonly used for measuring glucose in urine (range, 1–50 g/l) as previously described (Viana et al. [2012]). Samples were periodically taken after glucose exhaustion, and the fructose concentration was estimated by using the Nelson-Somogyi method (Nelson [1944]), as described previously (Fournier [2001]). A calibration curve was created by correlating the OD_620nm_ to the fructose concentration of standard solutions in the range of 0 to 2 g/l, using linear regression (R^2^ ≥ 0.99).

### Fermentation kinetics

The duration of the lag phase was quantified as the time obtained by extrapolating the tangent at the exponential part of the growth curve, back to the inoculum level1 (Swinnen et al. [2004]).

The time to the stationary phase was defined as the time necessary to reach the first of two equal OD values within a minimum interval of 5 h. The end of glucose fermentation was defined as the time when glucose was exhausted (0 g/l). The end of fermentation was defined as the time when the fructose concentration dropped below 2 g/l. These points were used to estimate the alcoholic fermentation times (in h) and the time necessary to ferment glucose (AF1) and fructose (AF2), excluding the lag phase.

### Reproducibility of the results

All experiments were performed at least 3 times. Mean values or results of typical experiments are presented, as indicated.

## Results

### Strain S288C is a good laboratory model for white grape must fermentation

**Figure 1:**
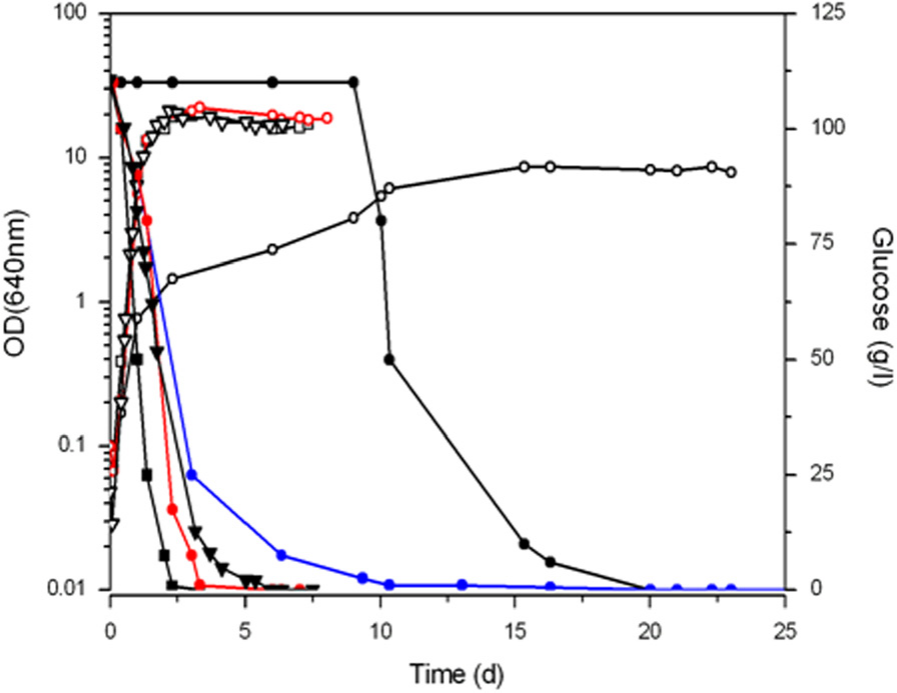
**Fermentation performances of*S. cerevisiae*ISA1000, S288C and BY4741 strains in natural grape must (NGM).** Open symbols indicate optical density for ISA1000 (**▽**), S288C (**□**) and BY4741 (**◯** and red circle symbol) strains, respectively. Glucose consumption is represented by closed symbols. Glucose was completely consumed by ISA1000 and S288C strains (▼ and ■), in NGM; for BY4741 strain glucose was completely consumed in NGM (●), in NGM with auxotrophic supplements (1×) (blue circle symbol) and in NGM with auxotrophic supplements (2×) (red circle symbol). All fermentations were performed at 25°C. Representative results of one of three independent experiments are shown. OD, optical density.

Recently, we characterized the fermentative performance of an enological yeast strain (*S. cerevisiae* ISA1000) during fermentation of NGM from the Portuguese white grape variety Arinto (Viana et al. [2012]). In the present work, we studied the behavior of the laboratory haploid yeast strain *S. cerevisiae* S288C in NGM, comparing fermentation parameters of this strain with those obtained for the enological yeast *S. cerevisiae* ISA1000 under the same conditions. Figure 1 shows the growth curves and glucose consumptions for the two strains. Table 1 presents the corresponding macrokinetic parameters (i.e., lag-phase, doubling time, final OD, AF1 and AF2).

**Table 1:** Macrokinetic parameters for fermentations of NGM, RB-SGM and ISA-SGM by ISA1000, S288C and BY4741 strains

**Medium**	**Supp. additionª**	**Strain**	**Lag-phase (h)**	**Doubling time (h)**	**Final OD**	**Minimum [Glucose] (g/L)**	**AF1**^**b**^**(h)**	**AF2**^**b**^**(h)**	**Complete fermentation**
NGM	--	ISA1000	<2	2.4 ± 0.18	21.2 ± 0.76	0	142	166	**YES**
	--	S288C	<2	3.3 ± 0.12	20.4 ± 1.87	0	70	150	**YES**
	--	BY4741	4 ± 0.67	7.8 ± 0.11	8.8 ± 0.43	0	476	530	**YES**
	1x	BY4741	<2	3.9 ± 0.18	15.3 ± 0.83	0	476	528	**YES**
	2x	BY4741	<2	3.3 ± 0.20	19.6 ± 1.42	0	145	152	**YES**
RB-SGM	--	S288C	40 ± 0.84	3.4 ± 0.21	10.4 ± 0.88	35	>570	>570	**NO**
ISA-SGM	--	ISA1000	3 ± 0.75	2.6 ± 0.15	23.4 ± 0.64	0	106	150	**YES**
	2x	ISA1000	4 ± 0.74	1.9 ± 0.12	22.6 ± 0.74	0	105	140	**YES**
	--	S288C	9 ± 1.71	3.4 ± 0.21	24.2 ± 1.61	0	159	183	**YES**
	2x	S288C	8 ± 0.71	2.3 ± 0.11	23.3 ± 0.97	0	150	165	**YES**
	2x	BY4741	9 ± 0.62	3.6 ± 0.26	23.6 ± 0.83	0	159	300	**YES**

Data represent the average of data from at least two independent cultures.

Standard deviation calculated from duplicate experiments (± standard deviation).

^a^Concentration of auxotrophic supplements approximately corresponding to those described by Pronk ([2002]).

^b^AF1 (Alcoholic Fermentation for Glucose) and AF2 (Alcoholic Fermentation for Fructose) are the time for glucose and fructose fermentation excluding the lag-phase, respectively.

Both ISA1000 and S288C were able to ferment NGM efficiently up to the maximum legal limit for reducing sugars. Fermentation curves for the enological and laboratory strains showed that S288C grew more slowly during the exponential phase than ISA1000, with a specific growth rate of (0.21 ± 0.0079)/h compared (0.31 ± 0.0232)/h to ISA1000. Nevertheless, S288C fermented glucose more rapidly than ISA1000; the time required for S288C to exhaust glucose (AF1) was 70 h, whereas ISA1000 took 142 h. The duration of fructose fermentation (AF2) was similar for both strains (150 and 166 h, respectively) (Table 1). As expected, both strains fermented fructose at a slower rate than glucose.

### Improved SGM (ISA-SGM) leads to successful wine fermentations by S288C and ISA1000

**Figure 2:**
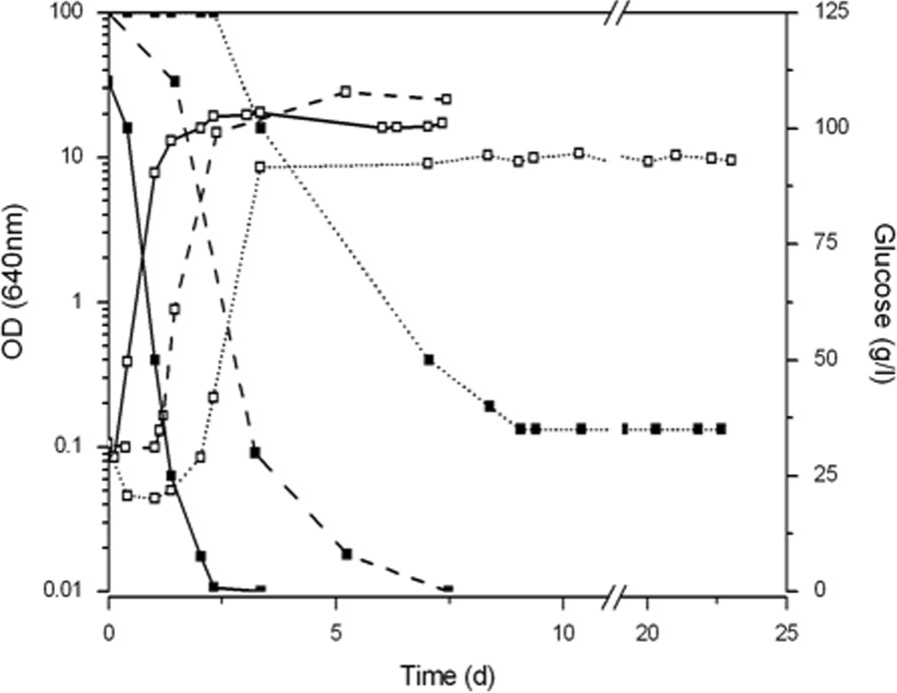
**Fermentation performances of*S. cerevisiae*S288C strains in NGM, in RB-SGM and in ISA-SGM.** Open squares (**□**) indicate optical density for S288C strain in NGM (solid line), in RB-SGM (dot line) and in ISA-SGM (dash line). Glucose (■) was completely consumed by S288C strain in NGM and in ISA-SGM, but in RB-SGM fermentation got stucked, lasting 35 g/l of residual sugar in the medium. All fermentations were performed at 25°C. Representative results of one of three independent experiments are shown. OD, optical density.

To create a reliable SGM formulation for use in fermentations by laboratory strains, we modified a basal SGM formulation (RB-SGM) (Rossouw and Bauer [2009]) by considering suggestions from Marullo et al. ([2004]) and Albertin et al. ([2011]), together with the data on the composition of NGMs from Ribereau-Gayon et al. ([2006]). The fermentative performance of S288C was evaluated in RB-SGM with the addition of 50 ppm SO_2,_ anaerobic growth factors, and varying amounts and combinations of malic (0, 3, and 6 g/l), citric (0, 0.3, and 6 g/l), and tartaric acids (0 and 3 g/l). S288C fermenting basal RB-SGM presented the same maximum growth rate as S288C fermenting NGM; however, it also had a significantly longer lag phase and a 49% reduction in the stationary phase biomass. S288C was not capable of completing the RB-SGM fermentation, leaving 35 g/l of residual glucose (Table 1 and Figure 2). Under these conditions, the fermentation was stuck. Among all of the tested formulations (results not shown), only the combination of 3 g/l malic acid, 0.3 g/l citric acid, and 3 g/l tartaric acid allowed S288C to complete synthetic must fermentation up to must dryness (Figure 2). These values are close to those used by Wang et al. [2003].

The simultaneous introduction of small amounts of malic, citric, and tartaric acids along with anaerobic growth factors and SO_2_ led to a fermentative performance that was comparable to the fermentative performance of S288C in NGM (see values of AF1 and AF2 in Table 1). Therefore, we selected this formulation as our standard SGM, and designated it as ISA-SGM, for ***I***nstituto ***S***uperior de ***A***gronomia-***S***ynthetic ***G***rape ***M***ust. Additional file 1 describes in detail the composition of ISA-SGM.

**Figure 3:**
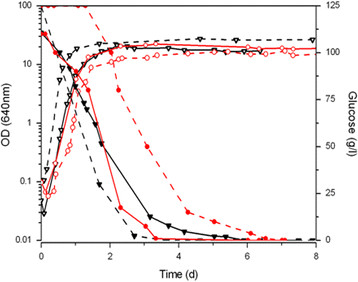
**Fermentation performance of*S. cerevisiae*ISA1000 and BY4741 strains in NGM and in ISA-SGM.** Open symbols indicate optical density for ISA1000 strain (**▽**) in NGM (black solid line) and in ISA-SGM (black dash line); for 2x auxotrophic supplemented NGM (red solid line) and ISA-SGM (red dash line) BY4741 growth is represented as (red circle symbol). Glucose was completely consumed for both strains (▼, red circle symbol) and in all media (solid and dash lines). All fermentations were performed at 25°C. Representative results of one of three independent experiments are shown. OD, optical density.

During S288C fermentation in both ISA-SGM and NGM, more than 50% of the yeast cells were viable (results not shown). This finding is similar to our previous result obtained for enological strain ISA1000 in NGM (Viana et al. [2012]). We compared the fermentative performance of the commercial wine strain ISA1000 in ISA-SGM with our previous results of the ability of ISA1000 to ferment NGM (Viana et al. [2012]). As shown in Figure 3, the growth curves were comparable in both types of media. The stationary phase was reached with a similar final biomass within a similar timeline (Table 1).

Although ISA-SGM contained 15 g/l more of each sugar, the commercial strain was still able to ferment the sugars up to must dryness. Fermentation times were shorter for glucose in ISA-SGM (AF1 of 106 and 142 h for ISA-SGM and NGM, respectively) and were comparable for fructose (AF2 of 150 and 166 h for ISA-SGM and NGM, respectively) (Table 1). For ISA1000, we observed a cell viability of 50% at the end of ISA-SGM fermentation (results not shown), consistent with our previous observations in NGM (Viana et al. [2012]).

### Additional auxotrophic supplements improve fermentative performance of BY4741 in NGM and in ISA-SGM

To validate the utility of the S288C-derived auxotrophic strain BY4741 and its single deletion mutant library available at Euroscarf, we performed fermentation assays with the BY4741 strain under the same conditions as described for ISA1000 and S288C. We also investigated the role of auxotrophic supplementation in NGM fermentation. When we compared the fermentative performances of S288C and BY4741 in NGM, the results confirmed a clear effect of the addition of extra supplementation on BY4741 behavior. BY4741 demonstrated the best fermentative performance with increased concentrations of auxotrophic supplements. Figure 1 and Table 1 show the growth kinetics of BY4741 in NGM supplemented with uracil, histidine, leucine, and methionine at 1× and 2× the previously recommended concentrations for anaerobic fermentation (Pronk [2002]). Kinetics was monitored by OD_640nm_ and by the sugar concentration, and was compared to S288C. Use of NGM without additional supplementation permitted complete must fermentation by BY4741; however, the BY4741 growth and sugar consumption parameters were severely affected. The lag-phase time was considerably increased, and the doubling time was decreased (specific growth rate for BY4741 was (0.089 ± 0.0012)/h, much lower than that for S288C). The final OD_640nm_ was only 43% of the OD_640nm_ obtained for S288C under the same conditions.

However, the addition of 2× the auxotrophic supplements resulted in a specific growth rate ((0.21 ± 0.013)/h) and final biomass (OD_640nm_ of 19.6) similar to those of S288C. With the auxotrophic supplements (2×), BY4741 took the same time to complete sugar fermentation (AF1 and AF2) as the enological strain *S. cerevisiae* ISA1000 (Table 1). As a control, strains ISA1000 and S288C were grown in the same medium: the inclusion of additional nitrogen sources did not disturbed the strains performance. Therefore, the slower fermentation of the auxotrophic strain was at least partially due to the auxotrophic mutations. Moreover, poor fermentation performance could be overcome by increasing the concentration of the appropriate supplements.

To validate the use of ISA-SGM in studies with the auxotrophic BY4741 strain, we compared the fermentative performances of this strain in ISA-SGM and in NGM. The concentrations of auxotrophic supplements were maintained at 2× the concentrations recommended for anaerobic fermentation (Pronk [2002]). As we observed for S288C, fermentation of both sugars up to dryness was achieved by BY4741 in supplemented ISA-SGM (Figure 3 and Table 1). Additionally, when we compared BY4741 fermentations in ISA-SGM and NGM (both 2× supplemented), glucose was consumed in a similar time frame for both media (AF1) (Figure 3 and Table 1), and a similar 50% reduction in cell viability was observed (results not shown). However, some differences were still evident, as both the lag-phase and AF2 were significantly longer in ISA-SGM (Table 1).

## Discussion

Natural grape must is a very complex and variable medium that has a great impact on the efficiency of yeast fermentation. The concentrations of sugar, nitrogen, and micronutrients change from season to season, and the composition of NGM depends on the grape variety, geographic and climate factors, viticulture practices, and the degree of fruit ripeness at harvest. The diversity of winemaking practices also contributes to the variability of grape must composition and fermentation conditions. Taken together, these factors make it difficult to compare fermentation performance between strains and studies. To achieve better experimental reproducibility, researchers frequently use SGMs with defined compositions when studying fermentation performance.

An increasing demand for new strains to optimize wine production has led to the development of yeast improvement programs (Giudici et al. [2005]; Verstrepen et al. [2006]). The selection of yeast strains with interesting features requires knowledge of yeast genetics and physiological diversity. To understand the fermentative behavior of yeast while fermenting grape must, the use of single deletion mutant collections fermenting defined SGMs may be a powerful approach. Yet, it is often difficult to extrapolate the behavior of laboratory strains under laboratory conditions to the behavior of commercial wine strains fermenting NGM under winery conditions. It is therefore mandatory a previous step of validation of both strain and must composition.

In this work, we aimed to test and validate the use of the parental *S. cerevisiae* S288C and its isogenic auxotrophic derivative BY4741 as laboratory models for wine fermentation studies. These strains presented fermentative performances similar with the known practical properties of the enological strain ISA1000 in NGM. We also designed a new SGM formulation (ISA-SGM), by adding glucose and fructose in equal amounts (125 g/l) and 50 parts per million (ppm) sulfur dioxide by adding potassium metabisulfite (corresponding to standard enological treatment), and we optimized the concentrations of malic acid (3 g/l), citric acid (0.3 g/l), and tartaric acid (3 g/l), to mimic the normal concentrations of those acids commonly found in must at grape maturity (Ribereau-Gayon et al. [2006]). We concluded that meaningful enological wine fermentation studies performed with S288C-derived laboratory strains could use ISA-SGM, as long as the proper concentrations of auxotrophic supplements are provided. When we compared the growth and fermentation properties of laboratory strain S288C in NGM with previous results obtained for the enological *S. cerevisiae* ISA1000 strain (Viana et al. [2012]), both strains presented similar fermentative performances (Figure 1). It is likely that the NGM variability explains the discrepancies with previous results for the S288C strain, which had been described as an intrinsically poor fermenter (Harsch et al. [2010]; Pizarro et al. [2007]).

To avoid the variability of NGMs and the unpredictable behavior of laboratory strains during their fermentation, several SGMs have been designed (Harsch et al. [2010]; Rossouw and Bauer [2009]). However, as far as we know, none of these formulations has been completely successful. Moreover, most do not include SO_2_, which is common in standard enological practices. Therefore, we sought to develop a SGM to simulate the main stress factors found in NGM, including low vitamin concentration, high glucose and fructose concentrations, and the presence of weak acids. We mimicked winemaking practices by adding SO_2_ (final concentration of 50 ppm).

We determined that ISA-SGM was a good model of NGM, as measured by yeast growth and sugar consumption. Certainly, an important role is played by the increase of concentrations of malic, citric, and tartaric acids, which were closer to those commonly found in NGM. At lower concentrations of these acids, glucose was not exhausted and the fermentation became stuck. Only the combination of 3 g/l malic acid, 0.3 g/l citric acid, and 3 g/l tartaric acid led to synthetic must fermentation up to dryness (<2 g/l residual sugar). Previous results may explain the effect of these weak acids. For example, it was shown that the presence of acetic acid causes an increase in glycolytic flux in yeast (Pampulha and Loureiro-Dias [2000]). In the presence of weak acids, the Pma1 H^+^-ATPase mainly guarantees proton homeostasis. The additional H^+^ outflow required for maintenance of the intracellular pH in the presence of weak acids dissipates extra ATP, decreasing cytosolic ATP pool. It has been reported that a decrease of the ATP levels in the cytoplasm stimulates the glycolytic flux (Larsson et al. [1997]). It is conceivable that weak acids, which promote ATP consumption, contribute to increased sugar consumption rates and allow the SGM to reach dryness.

Because of its complex and variable nature, it is likely impossible to reproduce the composition of NGM completely. Despite the high performance in ISA-SGM, its composition does not match the complete composition of NGM. Particularly, it lacks the chemical precursors required for wine flavor (Styger et al. [2011]; Swiegers and Pretorius [2005]), which can affect yeast performance.

Using auxotrophic strains is convenient for detailed molecular studies of yeast fermentation, but their auxotrophy is a major drawback for the analysis of growth kinetics and stress effects. As a result, it can be difficult to translate results from the laboratory to an industrial setting (Pronk [2002]). Additionally, the type of auxotrophic mutations, level of supplementation required, and particular growth medium used all have large effects on yeast growth kinetics. Often, the auxotrophic nutrient becomes limiting for growth (Bauer et al. [2003]; Cohen and Engelberg [2007]), inducing a physiological state that is different from that of cells whose growth is limited by standard biological nutrients, such as carbon, nitrogen, or phosphate (Brauer et al. [2008]), or by metabolite toxicity. Despite these concerns, many of the genetic tools available for yeast have been constructed in auxotrophic strains.

While fermenting non-supplemented NGM, BY4741 fermented the must up to sugar exhaustion. Nevertheless, in non-supplemented NGM, BY4741 showed a moderate decrease in biomass levels and a significant increase in sugar fermentation times compared to the parental prototrophic S288C strain (Table 1). Experiments comparing the growth curves of the prototrophic *S. cerevisiae* S288C with the isogenic BY4741 auxotrophic mutant have reported similar results to ours for mineral and YPD media (Paciello et al. [2009]).

Even NGM, a complex rich medium, did not fully compensate for the auxotrophic growth deficiencies of the BY4741 strain, which was unable to grow to its maximum biomass and accomplish an efficient wine fermentation in NGM. To overcome this deficiency, we hypothesized that the concentration of one or several essential supplements required for auxotrophy (uracil, leucine, histidine, and methionine) were present in concentrations below those required for optimized biomass production. Considering the expected maximum biomass reached during NGM fermentation (Viana et al. [2012]) and the biomass content on each of the four supplements (Pronk [2002]), we calculated minimum amounts to be added of 60 mg/l uracil, 40 mg/l methionine, 200 mg/l leucine, and 50 mg/l histidine. These values are close to those recommended by Pronk ([2002]), considering that all of the supplements were used for biomass accumulation and not for further metabolism.

Assays were performed in NGM supplemented with the minimum and twice the amounts of these calculated concentrations. The minimum level of supplementation improved the fermentation parameters of BY4741. However, although the final biomass was closer to that of S288C in NGM, the sugar consumption by BY4741 was still significantly slower than that of S288C (Table 1, Figure 1). Supplementing NGM with the highest concentrations of auxotrophic supplements (120 mg/l uracil, 80 mg/l methionine, 400 mg/l leucine, and 100 mg/l histidine) further increased the final biomass of BY4741 to levels of the prototrophic parental strain S288C and the enological commercial strain ISA1000 fermenting NGM without auxotrophic supplements (Table 1, Figure 1). In addition, the AF1 and AF2 fermentation times in the 2× supplemented NGM were reduced to approximately 30% of the time required to ferment NGM without extra supplementation. These fermentation times were similar to those presented by S288C and ISA1000 fermenting NGM without extra supplementation.

In conclusion, we have developed a synthetic grape must, ISA-SGM, in which a prototrophic laboratory strain and an auxotrophic strain (appropriately supplemented) present fermentative profiles similar to that of a commercial wine strain in natural grape must. ISA-SGM can be used as a new tool for BY mutants in the detailed assessment of the alcoholic fermentation process under conditions close to those found in wineries. This new formulation may provide a sound foundation to extract relevant physiological conclusions on enological molecular yeast traits.

## Competing interests

The authors declare that the research was conducted in the absence of any commercial or financial relationships that could be construed as potential conflicts of interest.

## Additional file

## Supplementary Material

Additional file 1: **Chemical composition of*I*nstituto*S*uperior de*A*gronomia –*S*ynthetic*G*rape*M*ust (ISA-SGM).** pH was adjusted to 3.3 using NaOH. Concentrated solutions of each compound were prepared, filtered through 0.22-μm nitrocellulose membranes (Millipore filter, type GSWP), and added in adequate amounts before inoculation. Click here for file
