# Supplementary material for: Efficient fermentation of an improved synthetic grape must by enological and laboratory strains of Saccharomyces cerevisiae
Source: AMB Express. 2014 Apr 1;4:16. doi: 10.1186/s13568-014-0016-0 (PMC4052690; doi:10.1186/s13568-014-0016-0)
Supplement: Additional file 1: — Chemical composition ofInstitutoSuperior deAgronomia –SyntheticGrapeMust (ISA-SGM). pH was adjusted to 3.3 using NaOH. Concentrated solutions of each compound were prepared, filtered through 0.22-μm nitrocellulose membranes (Millipore filter, type GSWP), and added in adequate amounts before inoculation. [file s13568-014-0016-0-S1.doc]

**Additional file 1**

Chemical composition of Instituto Superior de Agronomia – Synthetic Grape Must (ISA-SGM). pH was adjusted to 3.3 using NaOH. Concentrated solutions of each compound were prepared, filtered through 0.22-µm nitrocellulose membranes (Millipore filter, type GSWP), and added in adequate amounts before inoculation.

| **Chemical compound** | **Formula** | **Quantity** | **Unit** |
| --- | --- | --- | --- |
| Glucose | C_6_H_12_O_6_ | 125 | g/l |
| Fructose | C_6_H_12_O_6_ | 125 | g/l |
| Calcium pantothenate | C_9_H_17_NO_5_ | 1.5 | mg/l |
| Thiamine HCl | C_12_H_18_Cl_2_Na_4_OS,HCl | 0.250 | mg/l |
| Pyridoxine HCl | C_8_H_12_ClNO_3_ | 0.250 | mg/l |
| Biotin | C_10_H_16_N_2_O_3_S | 0.003 | mg/l |
| Myo-inositol | C_6_H_12_O_6_ | 20 | mg/l |
| Nicotinic acid | C_6_H_5_NO_2_ | 2 | mg/l |
| Potassium dihydrogen phosphate | KH_2_PO_4_ | 750 | mg/l |
| Potassium sulfate | K_2_SO_4_ | 500 | mg/l |
| Magnesium sulfate | MgSO_4_.7H_2_O | 250 | mg/l |
| Calcium chloride | CaCl_2_.2H_2_O | 155 | mg/l |
| Sodium chloride | NaCl | 200 | mg/l |
| Manganese sulfate | MnSO_4_.H_2_O | 4 | mg/l |
| Zinc sulfate | ZnSO_4_ | 4 | mg/l |
| Copper sulfate | CuSO_4_.5H_2_O | 1 | mg/l |
| Potassium iodide | KI | 1 | mg/l |
| Cobalt (II) chloride | CoCl_2_.6H_2_O | 0.4 | mg/l |
| Boric acid | H_3_BO_3_ | 1 | mg/l |
| Sodium molybdate | NaMoO_4_.2H_2_O | 1 | mg/l |
| Ammonium chloride | NH_4_Cl | 460 | mg/l |
| L-proline | C_5_H_9_NO_2_ | 612.6 | mg/l |
| L-glutamine | C_5_H_10_N_2_O_3_ | 505.3 | mg/l |
| L-arginine | C_6_H_14_N_4_O_2_ | 374.4 | mg/l |
| L-tryptophan | C_11_H_12_N_2_O_2_ | 179.3 | mg/l |
| L-alanine | C_3_H_7_NO_2_ | 145.3 | mg/l |
| L-glutamic acid | C_5_H_9_NO_4_ | 120.4 | mg/l |
| L-threonine | C_3_H_7_NO_3_ | 759.2 | mg/l |
| L-serine | C_4_H_9_NO_3_ | 78.5 | mg/l |
| L-leucine | C_6_H_13_NO_2_ | 48.4 | mg/l |
| L-aspartic acid | C_4_H_7_NO_4_ | 44.5 | mg/l |
| L-valine | C_5_H_11_NO_2_ | 44.5 | mg/l |
| L-phenylalanine | C_9_H_11_NO_2_ | 37.9 | mg/l |
| L-isoleucine | C_6_H_13_NO_2_ | 32.7 | mg/l |
| L-histidine | C_6_H_9_N_3_O_2_ | 32.7 | mg/l |
| L-methionine | C_5_H_11_NO_2_S | 31.4 | mg/l |
| L-tyrosine | C_9_H_11_NO_3_ | 18.3 | mg/l |
| L-glycine | H_2_NCH_2_COOH | 18.3 | mg/l |
| L-lysine | C_6_H_14_N_2_O_2_ | 17 | mg/l |
| L-cysteine | C_3_H_7_NO_2_S.HCl.H_2_O | 13.1 | mg/l |
| Ergosterol | C_28_H_44_O | 15 | mg/l |
| Sodium oleate | C_18_H_33_O_2_Na | 5 | mg/l |
| Malic acid (DL-) | C_4_H_6_O_5_ | 3 | g/l |
| Citric acid | C_6_H_8_O_7_ | 0.3 | g/l |
| Tartaric acid (L+) | C_4_H_6_O_6_ | 3 | g/l |
| Potassium metabisulfite | K_2_O_5_S_2_ | 100 | mg/l |
